# Laparoscopic versus open radical prostatectomy in high prostate volume cases: impact on oncological and functional results

**DOI:** 10.1590/S1677-5538.IBJU.2015.0385

**Published:** 2016

**Authors:** Sciarra Alessandro, Gentilucci Alessandro, Cattarino Susanna, Innocenzi Michele, Di Quilio Francesca, Fasulo Andrea, Magnus Von heland, Gentile Vincenzo, Salciccia Stefano

**Affiliations:** 1 Dipartimento di Urologia - Unità della prostata, Università La Sapienza, Roma, Italia; 2 Istituto Nazionale Italiano di Statistica - Ricercatore di Statistica, Roma, Italia

**Keywords:** Laparoscopy, Prostatic Neoplasms, Prostate, Prostatectomy, Surgical Procedures, Operative

## Abstract

**Background and objective:**

To prospectively compare the laparoscopic versus open approach to RP in cases with high prostate volume and to evaluate a possible different impact of prostate volume.

**Materials and Methods:**

From March 2007 to March 2013 a total of 120 cases with clinically localized prostate cancer (PC) and a prostate volume>70cc identified for radical prostatectomy (RP), were prospectively analyzed in our institute. Patients were offered as surgical technique either an open retropubic or an intraperitoneal laparoscopic (LP) approach. In our population, 54 cases were submitted to LP and 66 to open RP. We analyzed the association of the surgical technique with perioperative, oncological and postoperative functional parameters.

**Results:**

In those high prostate volume cases, the surgical technique (laparoscopic versus open) does not represent a significant independent factor able to influence positive surgical margins rates and characteristics (p=0.4974). No significant differences (p>0.05) in the overall rates of positive margins was found, and also no differences following stratification according to the pathological stage and nerve sparing (NS) procedure.

The surgical technique was able to significantly and independently influence the hospital stay, time of operation and blood loss (p<0.001). On the contrary, in our population, the surgical technique was not a significant factor influencing all pathological and 1-year oncological or functional outcomes (p>0.05).

**Conclusions:**

In our prospective non randomized analysis on high prostate volumes, the laparoscopic approach to RP is able to guarantee the same oncological and functional results of an open approach, maintaining the advantages in terms of perioperative outcomes.

## INTRODUCTION

Laparoscopic (LP) prostatectomy has become a common treatment option for patients with localized prostate cancer (PC). Non randomized studies compared the laparoscopic with the open approach for radical prostatectomy (RP) (1–4). A large population-based study found similar oncological and functional results comparing the two techniques (5), whereas other investigations have found significant differences (6, 7). Several aspects could contribute to these different results, such as selection of patients, methods of analysis and also surgeon/center experience in RP. The choice of the surgical technique for localized PC is in part based on personal preference. However, some considerations such as age, preoperative status, staging and grading may influence choice of modality. Each patient presents with a unique set of characteristics that could influence the technique of RP regardless of the approach (1–4). Prostate volume is an important consideration for surgery, particularly when patients have large glands (8). In some studies smaller glands have been associated with high grade disease, more advanced stage and higher rate of positive surgical margins (9–11). On the other hand the difficulty of dissecting a large prostate gland has been recognized since the early history of RP (12). The surgical and technical impact of prostate size could be more relevant in the LP (13), probably because larger prostates decrease visualization of the surgical field when performing the laparoscopic approach.

The objective of this study was to prospectively compare the laparoscopic versus open approach to RP in cases with high prostate volume and to evaluate a possible different impact of prostate volume either in terms of oncological or functional results.

## MATERIALS AND METHODS

### Study design and population

From March 2007 to March 2013 a total of 120 cases with clinically localized PC and a prostate volume>70cc identified for RP, were prospectively analyzed in our institute. This sample was part of a population of 296 cases with clinically localized PC selected for RP. All patients provided informed consent and the study was approved by our institutional review board.

Inclusion criteria were an histological diagnosis of PC at biopsy, clinically defined T1c-T2N0M0 stage and a prostate volume>70cc. All cases accepted the surgical option as primary treatment. Exclusion criteria were prior surgery at bladder or prostate level, neoadjuvant treatments (hormonal or radiation therapies), contraindications for surgical treatment.

Patient demographics, intraoperative and postoperative parameters were prospectively collected and analyzed. In particular prostate volume was measured preoperatively by magnetic resonance imaging (MRI) using the widely recognized prostate ellipsoid method (11) and only cases with values>70cc were included in this analysis. Preoperatively all cases were submitted to a multiparametric MRI for staging and decision making for a nerve sparing procedure.

Patients were offered as surgical technique either an open retropubic or an intraperitoneal laparoscopic approach. In our population of 120 cases, 54 were submitted to LP and 66 to open RP. The preoperative assessment of all patients included detailed patient history, clinical examination, serum prostate specific antigen (PSA) measurement, prostate volume determination at MRI, prostate biopsy findings, Gleason score assessment, imaging studies (MRI and bone scan or PET-CT scan). Only cases with clinical T2N0M0 staging were included.

### Surgical Technique

All cases were submitted to RP by a surgeon with 10 years of experience with open RP (more than 100 cases performed) and 5 years with LP (more than 50 cases performed).

The choice between the open or laparoscopic approach was discussed with the patient but no specific selection was performed regarding the surgical approach. Fifty-five cases were submitted to a standard open retropubic RP as previously described (13), and 65 to a standard intraperitoneal laparoscopic RP as previously described (14). In none of these cases a lymph-node dissection was performed. The decision for a intrafascial nerve sparing (NS) (monolateral or bilateral) procedure was homogeneously considered in all cases regardless on the surgical technique and included preoperative status of the patient, biopsy findings and imaging (MRI) results. In particular a bilateral intrafascial NS procedure was performed in sexually active cases with a PSA level<10ng/ml and a Gleason score<7or only one core with Gleason score 7 and clinical T1c-T2a tumors. To perform a NS procedure, we used a retrograde approach and we used only clips instead of the energy for the coagulation in the dissection phase. We did not use specific surgical procedures to improve continence but a standard anastomosis has been performed. The operative parameters prospectively considered in the study were surgical technique, NS procedures, estimated blood loss, operative time and intraoperative complications.

### Pathological examination

All surgical specimens were processed by one referent uro-pathologist. All specimens were inked and fixed in buffered formalin. The prostate and seminal vesicles were entirely sectioned at 3-mm intervals. The presence of malignant glands in direct contact with the inked surface was considered as constituting positive surgical margins (SM). The pathological parameters included in our analysis were prostate weight, tumor volume, Gleason score (high (2–6), moderate (7) and poor (8–10) differentiation according to the WHO consensus), SM status and localization, pathological stage.

### Postoperative evaluation and patient follow-up

The postoperative evaluation included hospital stay, time for catheter removal and complications. All cases underwent cystography at postoperative day 7 and the catheter was removed if no extravasation was recorded. Intraoperative and postoperative complications were assessed and graded according to the system described by Clavien (15). Follow-up visits were conducted at regular intervals. They included PSA monitoring, imaging examinations (MRI, bone scan or PET-CT scan), assessment of functional outcomes such as continence and erectile function.

Biochemical failure (BCF) was defined as postoperative PSA greater than 0.20ng/ml and confirmed.

The rate and time to recovery of continence was evaluated at regular intervals (1, 3, 6, 12 months) during a 1-year follow-up. Continence was defined as no pads use and no urinary leakage and subjective evaluation was made using the incontinence section of the ICS-male questionnaire (16).

The rate and time to recovery of erectile function (EF) was assessed using the IIEF-5 questionnaire during a 1-year follow-up (3, 6, 12 months). Patients who underwent a NS procedure during surgery were homogeneously submitted to erectile function rehabilitation using tadalafil 5mg/daily. Potency was defined as erections sufficient for penetration with or without phosphodiesterase inhibitors.

We also evaluated the “trifecta” (undetectable PSA, continence and potency) and “pentafecta” (trifecta plus no postoperative complications and negative surgical margins) rates in the two groups (17, 18).

### Statistical analysis

All parameters prospectively analyzed in the study are showed in Table-1 and presented as number of cases, mean±SD, median and range. For comparison between the two groups (Group 1=LP and Group 2=open RP) Student's t test and chi-squared test were used. An analysis of variance was used to compare continuous variables among the Groups. The association of the surgical technique with all other intraoperative and postoperative variables was evaluated using an univariate and multivariate linear regression model. Two-tailed P values<0.05 were considered statistically significant. A R 3.1.1 statistical software was used.

**Table 1:** Patient demographics in the two groups (LP versus open). Results are presented as mean±sD (mean) and range or number (%) of cases.

Parameter		Group 1-Laparoscopic	Group 2- Open	P value
**Number of cases**		54	66	--
Prostate volume (mL)		78.39±4.47 (78);72-86	78.12±3.74(78);72-90	0.8585
Age (years)		64.76±4.39 (66);55-70	64.74±4.30 (65);52-70	0.8657
BMI		25.65± 4.39 (25.8);20.5-28.7	25.76±1.39 (25.4);22.4-28.7	0.8674
Preoperative PSA (ng/mL)		6.83±2.53 (7.5);2.5-16.8	6.57±.25 (7.2);2.8-15.8	0.8055
**Clinical stage**				0.2454
	T1c	22 (40.7)	25 (37.9)	
	T2a	9 (16.7)	13 (19.7)	
	T2b	16 (29.6)	18 (27.3)	
	T2c	7 (13.0)	10 (15.1)	
**Clinical Gleason score**				0.2657
	6	37 (68.5)	40 (60.6)	
	7	15 (27.8)	24 (36.4)	
	8	2 (3.7)	2 (3.0)	
**NS procedure**				0.2566
	No NS	20 (37.0)	26 (39.4)	
	Monolateral	8 (14.8)	(5 (7.6)	
Bilateral		26 (48.1)	35 (53.0)	
**D'Amico risk classes**				0.3570
Low risk		8 (14.8)	12 (18.1)	
Intermediate risk		44 (81.5)	52 (78.8)	
High risk		2 (3.7)	2 (3.0)	

## RESULTS

Patient demographics in the two groups (LP versus open) are described in Table-1. All patients were stratified according to D’Amico risk classification. Mean prostate volume was 78.39±4.47ml and 78.12±3.74ml respectively in Groups 1 and 2 (p>0.05). In our population there was a significant correlation between preoperative prostate volume and RP specimen weight (coefficient 0.8980; p<0.001).

### Perioperative outcomes

Comparison in perioperative outcomes between laparoscopic and open procedures is showed in Table-2.

**Table 2:** Comparison of perioperative outcomes between laparoscopic and open procedures. Results are presented as mean ± SD (mean) and range or number (%) of cases.

Parameter	Group1- Laparoscopic	Group2- Open	P vale
Postoperative hospital stay (days)	4.35±5.54 (4);3-12	5.54±1.41 (5);4-14	<0.001
Operation time (minutes)	188.51±27.50 (190); 150-240	152.28±27.44 (170);100-200	<0.001
Blood loss (mL)	366.67±142.75 (400);100-700	572.73±174.13 (600);300-1000	<0.001
Transfusion rate	4 (7.4)	18 (27.3)	<0.001
Catheterization time (days)	7.65±2.11 (10);7-21	8.61±2.35 (10);7-21	0.02
Postoperative complications (Clavien score)			0.950
I	2 (3.7)	4 (6.1)	
II	3 (5.5)	3 (4.5)	
III	1 (1.8)	1 (1.5)	

Mean total operation time was significantly higher (p<0.001) in the laparoscopic (188.51±27.50 min) than in the open (152.28±27.44 min) group. Estimated mean blood loss and transfusion rates were both significantly (p<0.001) lower in Group 1 (366.67±142.75ml and 7.4% respectively) than in Group 2 (572.73±174.13ml and 27.3% respectively). Mean postoperative hospital stay was significantly lower (p<0.001) in Group 1 (4.35±5.54 days) than in Group 2 (5.54±1.41 days). Also, mean Foley catheterization time was significantly lower (p=0.02) in Group 1 (7.65±2.11 days) than in Group 2 (8.61±2.35 days).

No intraoperative complications were experienced in both groups and in all cases the procedure (laparoscopic or open) was successfully concluded. As classified according to the Clavien system, the rates of postoperative complication were very similar in the two groups (p=0.95). In particular no grade IV complications were experienced in both groups.

### Pathological outcomes

Pathological findings in the two groups after RP are showed in Table-3. The percentage of pT3 cases (18.5% and 19.7% respectively in Group 1 and 2) and the distribution of Gleason scores were similar (p>0.05) in the two groups.

**Table 3:** Comparison of pathological outcomes between laparoscopic and open procedures. Results are presented as mean ± SD (mean) and range or number (%) of cases.

Parameter		Group 1- Laparoscopic	Group 2- Open	P value
**pT stage**				0.4542
	pT2	44 (81.5)	53 (80.3)	
	pT3a	9 (16.7)	11 (16.7)	
	pT3b	1 (1.8)	2 (3.0)	
**Gleason score**				0.4762
	6	32 (59.2)	34 (51.5)	
	7	20 (37.0)	30 (45.4)	
	8	2 (3.7)	2 (3.0)	
**Positive surgical margins**				0.0846
	**Total number of cases**	4 (7.4)	7 (10.6)	
	Single	4 (7.4)	6 (9.1)	
	Multiple	0 (0)	1 (1.5)	
	Apical	3 (75.0)	5 (71.4)	
	Lateral	1 (25.0)	2 (28.6)	
	Basal	0 (0)	0 (0)	

No significant differences (p>0.05) in the overall rates of positive margins was found, and similarly also after stratification according to pathological stage and NS procedure. In particular in pT3 cases or in NS procedures, the rate of positive margins was similar (p>0.05) both groups (pT3 cases: Group 1=30.0% and Group 2=38.5%; NS procedure: Group 1=5.9% and Group 2=7.5%) (Table-4). Also regarding the number and location of positive surgical margins, most of these were apical (75% in Group 1 and 71.4% in Group 2) and no significant variations between the two groups were found (p>0.05).

**Table 4:** Extracapsular (pT3) cases and NS procedures: distribution and characteristics of positive surgical margins in the two groups. Results are presented as mean ± SD (mean) and range or number (%) of cases.

Positive surgical margin Parameter		Group 1 - Laparoscopic	Group 2 - Open	P value
**Extracapsular (pT3) disease**				
	**Total number**	3/10 (30.0)	5/13 (38.5)	0.3447
	Single	3/10 (30.0)	4/13 (30.8)	0.7424
	Multiple	0 (0)	1/13 (7.7)	
	Apical	2/10 (20.0)	4/13 (30.8)	
	Lateral	1/10 (10.0)	1/13 (7.7)	0.4420
	Basal	0 (0)	0 (0)	
**NS procedure**				
	**Total number**	2/34 (5.9)	3/40 (7.5)	0.3285
	Single	2/34 (5.9)	2/40 (5.0)	0.5744
	Multiple	0(0)	1/40 (2.5)	
	Apical	1/34 (2.9)	1/40 (2.5)	
	Lateral	1/34 (2.9)	2/40 (5.0)	0.5230
	Basal	0(0)	0(0)	

The percentage and number of positive surgical margins statistically significantly correlated with preoperative PSA (only at univariate analysis in laparoscopic group: p=0.0006), pT stage (at univariate and multivariate analysis in both groups: p<0.0001), Gleason score (only at univariate analysis in both groups: p<0.05) (Table-5).

**Table 5:** Linear regression model for parameters association with positive surgical margin finding.

Univariate analysis				
	Group 1 – Laparoscopic		Group 2 – Open	
Parameter	Coefficient	P.value	Coefficient	P value
Preoperative PSA	0.0469	0.0006	0.0195	0.2561
pT stage	0.9772	<0.0001	0.9622	<0.0001
Gleason score	0.9687	<0.0001	0.1705	0.0279
NS procedure	-0.0230	0.7728	-0.0296	0.7160
Multivariate analysis				
Parameter	Parameter estimates	Std.Error	T value	Pr(>|t|)
pT stage	1.0771	0.1750	6.1523	<0.0001

### Oncological outcomes

All cases were followed-up for at least for 12 months. Mean time of postoperative follow-up was 35.11±11.62 (range 12-48) months and 37.82±10.15 (range 12-48) months respectively in Groups 1 and 2.

**Figure 1:**
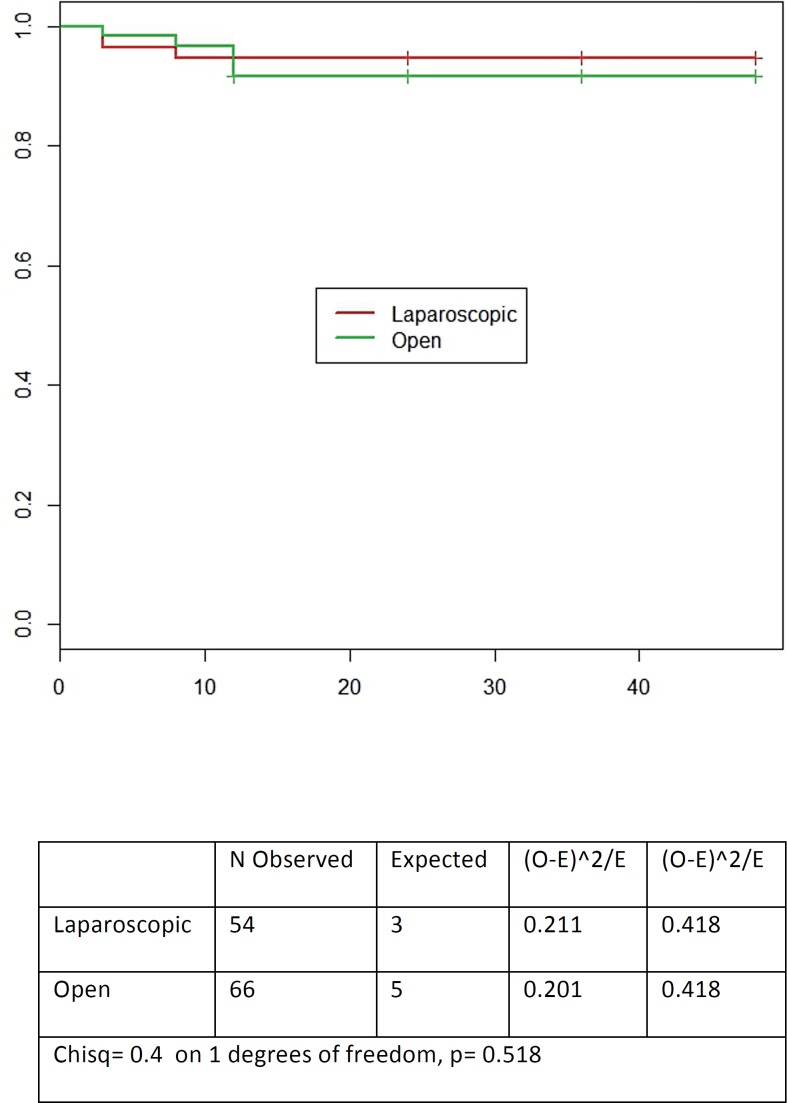
Estimated mean time for BC failure in laparoscopic versus open Rp.

No cases of deaths for any cause or clinical distant progression were reported. BC failure rates were similar (p>0.05) in the two groups (5.5% in Group 1 and 7.5% in Group 2). Mean time to BC failure is reported in Figure-1 (p=0.518). The risk of BF significantly correlated with preoperative PSA (only at univariate analysis and in the laparoscopic group: p=0.0048), pT stage (at univariate and multivariate analysis in both groups: p<0.05) and Gleason score (only at univariate analysis in both groups: p<0.05) (Table-6).

**Table 6:** Linear regression model for parameters association with BC failure.

Univariate analysis				
	Group 1- Laparoscopic		Group 2- Open	
Parameter	Coefficient	P-value	Coefficient	P-value
Preoperative PSA	0.1818	0.0048	0.0239	0.8735
pT stage	1.2222	0.0032	3.2727	<0.001
Gleason score	3.0234	0.0004	1.5666	0.0198
Positive surgical margins	0.4346	0.2356	1.4769	0.2623
Multivariate analysis				
Parameter	Parameter estimates	Std. Error	t value	Pr(>|t|)
pT stage	3.4661	0.8488	4.0834	0.0001

### Functional outcomes

Results in terms of continence and potency rates and regarding ICS and IIEF-5 questionnaires scores are reported in Table-7. After 12 months from surgery, continence rates were similar (p>0.05) between the two groups (98.1% and 98.5% respectively in Group 1 and 2). A significant difference in favor of the laparoscopic group (Group 1) was found at 3-and 6-month intervals (3-month: 88.9% and 75.7%; 6-month: 92.6% and 87.8% respectively in Group 1 and 2; p<0.05).

**Table 7:** Comparison in functional outcomes and trifecta and pentafecta results at 12 months after surgery, between laparoscopic and open procedures. Results are presented as mean ± SD (mean) and range or number (%) of cases.

Parameter		Group 1 - Laparoscopic	Group 2- Open	P value
Continence rate		53 (98.1)	65 (98.5)	0.7420
**ICS score**				0.020
	0	35 (64.8)	28 (42.4)	
	1	18 (33.3)	37 (56.1)	
	2	1 (1.8)	0(0)	
	3	0(0)	1 (1.5)	
Potency rate (in NS cases)		18/34 (52.9)	18/40 (45.0)	0.1845
IIEF-5 (in NS cases)		21.66±1.71 (22); 18-24	21.22±1.55 (22); 18-24	0.8540
Trifecta		18 (33.3)	18 (27.3)	0.1740
Pentafecta		16 (29.6)	17 (25.7)	0.3402

After 12 months of surgery, the percentage of potent cases was slightly higher in Group 1 (52.9%) than in Group 2 (45.0%) (p>0.05). These percentages are restricted in patients who had undergone a NS procedure (34 cases in Group 1 and 40 cases in Group 2).

### Trifecta and pentafecta outcomes

Overall, trifecta and pentafecta rates were 33.3% and 29.6% respectively in Groups 1 and 27.3% and 25.7% respectively in Group 2 (p>0.05) (Table-7). The mean characteristics of patients who reached a trifecta or pentafecta outcome in the two groups were similar: mean age 58.27±3.40 years, mean preoperative PSA 5.41±1.80ng/mL, pT2, Gleason score 6 or 7 and NS procedure.

Linear regression model analysis regarding the surgical approach

In Table-8 we summarized on which preoperative, pathological and postoperative outcomes a significant influence was determined by the surgical technique (laparoscopic versus open).

**Table 8:** Linear regression model analysis: estimation of surgical approach (laparoscopic versus open) influence on perioperative, pathological and postoperative outcomes.

Parameter		Parameter estimate	Std.Error	t value	Pr(>|t|)
**Postoperative hospital stay**					
Laparoscopic versus open		1.1936	0.2576	4.6323	<0.001
**Operation time**					
Laparoscopic versus open		36.2457	5.0405	7.1908	<0.001
**Blood loss**					
Laparoscopic versus open		206.0606	29.5049	6.9839	<0.001
**Catheterization time**					
	Laparoscopic versus open	0.9579	0.4117	2.3266	0.0216
**Postoperative complication rate (Clavier score)**					
	Laparoscopic versus open	0.0067	0.1110	0.0606	0.9517
**pT stage**					
	Laparoscopic versus open	0.0235	0.0867	0.2718	0.7862
**Gleason score**					
	Laparoscopic versus open	0.0707	0.1038	0.6806	0.4974
**Positive surgical margins**					
	Laparoscopic versus open	0.0471	0.0602	0.7820	0.4357
**BC failure**					
	Laparoscopic versus open	0.4528	0.3961	1.1432	0.2552
**Continence outcome**					
	Laparoscopic versus open	0.0033	0.0236	0.1421	0.8872
P**otency outcome**					
	Laparoscopic versus open	0.0794	0.1178	0.6739	0.5024
**Trifecta outcome**					
	Laparoscopic versus open	0.0606	0.0846	0.7162	0.4752
**Pentafecta outcome**					
	Laparoscopic versus open	0.0387	0.0825	0.4690	0.6398

In these high prostate volume cases, the surgical technique (laparoscopic versus open) does not represent a significant independent factor able to influence positive surgical margins rates and characteristics (p=0.4974). The surgical technique was able to significantly and independently influence the hospital stay, time to operation and blood loss (p<0.001). In our population, the surgical technique was not a significant factor influencing all pathological and 1-year oncological or functional outcomes (p>0.05). Also trifecta and pentafecta outcomes were not significantly determined by the surgical approach (p>0.05).

## DISCUSSION

As LP has been introduced for the surgical treatment of PC, there has been a continuous improvement in the understanding of technical factors that influence perioperative and postoperative outcomes. Previous series have investigated the effects of prostate volume on the outcomes of open RP and LP (19, 20). Larger prostates tend to influence operation time, blood loss and also pathological outcomes in terms of surgical margins. The surgical and technical impact of prostate size could be more relevant in the LP (13), probably because larger prostates decrease visualization of the surgical field when performing the laparoscopic approach. However, results in the literature are different (12, 19, 20) and not all found a significant influence related to prostate volume. Most of these studies (12, 19, 20) compared outcomes using the same surgical technique in different prostate volume categories. On the contrary, the objective of this study was to prospectively compare two different techniques, laparoscopic versus open RP, selecting only cases with high prostate volume and to evaluate a possible different impact of prostate volume (either in terms of oncological or functional results), depending on the surgical approach.

This is a prospective analysis focused only on large prostate volumes (median volume 78mL) considered for RP. Patients were not randomly assigned to LP versus open procedure. In the literature, mainly non randomized studies compared the laparoscopic with the open approach for RP (1–4). The choice between the open or laparoscopic approach was discussed with the patient but no specific selection was performed regarding the surgical approach. Large prostate volumes promote some difficulties during surgery, in particular regarding the preservation of the bladder neck and the apical dissection. The presence of a significant median lobe is frequent (65%) and it requires greater attention during bladder neck dissection. In our experience these situations can be successfully managed also during the laparoscopic procedure, increasing prostate gland traction.

Also, if this was not a randomized study, preoperative characteristics of patients in the two groups (LP versus open RP) were similar and not statistically different, either in terms of prostate volume, functional or oncological parameters.

Most of the differences between the two surgical approaches were found in terms of perioperative outcomes. Also, in larger prostate volumes, the LP is related to less blood loss and transfusion rates (p<0.001), less postoperative hospitalization and catheterization time (p<0.001) but longer operation times (p<0.001) when compared with the open RP. The laparoscopic approach does not increase the postoperative complication rate (p=0.95) when compared with open RP. In terms of functional postoperative outcomes the surgical approach in high prostate volumes does not significantly affect (p>0.05) continence and potency rates at 1-year follow-up. In a previous study (16) we underlined that prostate volume is a factor able to influence the time to recovery of continence after RP. In the present study, selecting only high prostate volumes, the percentage of continent cases were significantly higher in the LP group at 3-and 6-month intervals but not at 1-year interval between the two groups.

We focused our attention on pathological outcomes and on the possible impact of the surgical technique on positive surgical margins rates. Either using a laparoscopic or an open RP, pT stage represents the only significant and independent factor able to influence positive surgical margins and BF failure rates.

The surgical technique was not a significant factor able to influence positive surgical margins rates and characteristics. Using the LP approach in large prostate volumes, the rate and site of positive surgical margins were similar with those obtained using the open approach. Also in pT3 cases or NS procedures where the risk of positive margins could be higher, the LP approach does not influence results when compared to the open RP. Considering together functional and oncological parameters, the Trifecta and Pentafecta outcomes were similar in the two surgical groups.

## CONCLUSIONS

In our prospective non randomized analysis on high prostate volumes, the laparoscopic approach to RP is able to guarantee the same oncological and functional results of an open approach, maintaining the advantages in terms of perioperative outcomes (blood loss, hospital and catheterization time) and time to continence recovery.

## ABBREVIATIONS

LP: = Laparoscopic
PC: = Prostate cancer
RP: = radical prostatectomy
MRI: = Magnetic resonance imaging
SM: = Surgical margins
BCF: = Biochemical failure
BC: = Biochemical
PSA: = Prostate specific antigen
NS: = Nerve sparing
ED: = Erectile dysfunction
ICS: = International continence society
IIEF: = International index erectile function
